# Lumbar Fusion and Decompression in American Indian, Alaskan Native, Native Hawaiian, and Pacific Islander Populations: Healthcare Disparities in Spine Surgery

**DOI:** 10.7759/cureus.81409

**Published:** 2025-03-29

**Authors:** Mohammad F Khan, Saarang Patel, Dillon H Putzler, Avi N Albert, Hibbah I Khan, Ryan T Gensler, Maveric Abella, Jeffrey Hayashi, Frishan O Paulo, Julian L Gendreau, Janette Bow-Keola, Andrea Finlay, Derek F Amanatullah, Thomas Noh

**Affiliations:** 1 Neurosurgery, Indiana University School of Medicine, Indianapolis, USA; 2 Biological Sciences, Seton Hall University, South Orange, USA; 3 Neurosurgery, University of Hawaii John A. Burns School of Medicine, Honolulu, USA; 4 Neurosurgery, Meharry Medical College, Nashville, USA; 5 Neurosurgery, Georgetown University School of Medicine, Washington, D.C., USA; 6 Biomedical Engineering, Johns Hopkins University, Baltimore, USA; 7 Orthopedic Surgery, Stanford University School of Medicine, Stanford, USA

**Keywords:** american indian/alaskan native (ai/an), lumbar spine surgery, native hawaiian/pacific islander (nh/pi), postoperative outcomes, racial disparities, surgical equity

## Abstract

Introduction: Racial disparities in surgical outcomes are well documented, yet data on American Indian/Alaskan Native (AI/AN) and Native Hawaiian/Pacific Islander (NH/PI) populations remain limited. This study examines disparities in 30-day outcomes following lumbar decompression and fusion in these underrepresented groups.

Materials and methods: A retrospective analysis was conducted using the American College of Surgeons National Surgical Quality Improvement Program database (2017-2020). Patients undergoing lumbar decompression and fusion were identified via current procedural terminology codes. Multivariable logistic regression models adjusted for demographic and clinical factors assessed associations between race/ethnicity and postoperative outcomes, including readmission, complications, reoperation, and non-home discharge. Adjusted odds ratios (AOR) with 95% confidence intervals (CI) were reported.

Results: Among 113,340 patients, 0.38% (n=429) were AI/AN patients and 0.20% (n=229) were NH/PI patients. Compared to non-Hispanic White patients, AI/AN patients had higher odds of readmission (AOR: 1.023, 95% CI: 1.003-1.043, p=0.026) and complications (AOR: 1.030, 95% CI: 1.004-1.056, p=0.023). NH/PI patients had increased odds of readmission (AOR: 1.033, 95% CI: 1.006-1.062, p=0.018), major complications (AOR: 1.029, 95% CI: 1.007-1.051, p=0.009), and reoperation (AOR: 1.035, 95% CI: 1.014-1.057, p=0.001).

Conclusions: AI/AN and NH/PI patients face higher risks of adverse postoperative outcomes following lumbar spine surgery. Targeted interventions and increased inclusion in surgical disparities research are needed to improve equity in spine care.

## Introduction

The United States is racially diversifying, prompting a reevaluation of medical practices by physicians and policymakers to promote equity among minoritized races [1,2]. Spinal decompressions and fusions are common elective procedures often used to treat degenerative disease in the spine after the failure of non-surgical interventions [3]. With a growing elderly population and an increased need for these procedures, it is important to understand differences in outcomes for spine surgery for all racial populations [4].

There is increasing interest in addressing racial disparities in spine surgery; however, the vast majority of reports focus on non-Hispanic White, non-Hispanic Black, and Hispanic populations [5,6]. In contrast, few studies have evaluated the outcomes of lumbar spine surgery for Asian, American Indian, or Alaskan Native (AI/AN) and Native Hawaiian or Pacific Islander (NH/PI) patients [7]. A comprehensive literature review evaluating social determinants of health in spine surgery only found one study that specifically compared outcomes for Asian patients, and none specifically compared disparities in Native American or NH/PI patients [8]. A recent National Surgical Quality Improvement Project (NSQIP) database review of Anterior Cervical Discectomy and Fusion conducted by Elias et al. showed increased operative times in non-NH/PI and AI/AN Asian populations. However, this study did not evaluate NH/PI or AN populations [9].

There is a lack of quality research into health disparities for AI/AN Asian populations undergoing lumbar spine surgery. Thus, this study aimed to examine racial and ethnic disparities in underrepresented minoritized groups, with a specific focus on AI/AN and NH/PI populations, in lumbar spine surgery using the NSQIP database.

## Materials and methods

Study population

We included lumbar spine surgeries from 706 hospitals participating in the American College of Surgeons (ACS) NSQIP database from 2017 to 2020 [10]. The following current procedural terminology codes were used to identify procedures of interest: lumbar fusion at a single level (22533, 22558, 22612, 22630, 22633, 63052, 63053), lumbar fusion at multiple levels (22534, 22585, 22614, 22632, 22634, 63052, 63053), lumbar decompression (63005, 63012, 63017, 63042, 63044, 63047, 63048, 63056, 63057), and lumbar discectomy/microdiscectomy (63030, 63035, 62380) [10]. Data from each site are extracted by surgically certified reviewers who are intensively trained with continuing education courses to standardize data collection. Data consistency and reliability at each NSQIP site are evaluated by an inter-rater reliability audit program (1.6% disagreement rate) [11]. Variables obtained from the NSQIP database included patient demographics (age, BMI, operative time, female sex, active smoking status, American Society of Anesthesiologists (ASA) physical status classification score ≥3, one comorbidity, and multiple comorbidities), outcomes (readmission, postoperative complications, major complications, minor complications, reoperation, discharge destination other than home, mortality, and total hospital length of stay). Patients undergoing lumbar spine surgery who were a part of the AI/AN and NH/PI populations were included as we sought to specifically identify data for this population, given the limited amount of information available in the current state of the literature. Non-lumbar spine surgeries, emergency surgeries, and patients who were not part of the AI/AN and NH/PI populations were excluded. This study did not include charts with missing or null data sets (n=1,873, 1.63%).

Outcomes

The primary outcome was any adverse outcome within 30 days of surgery, which included readmission for any reason, any postoperative medical complication, major postoperative medical complications, minor postoperative medical complications, and reoperation for any reason. Secondary outcomes include non-home discharge destination and total hospital length of stay. A major complication was either a deep surgical site infection, organ or space surgical site infection, dehiscence, unplanned intubation, pulmonary embolism, ventilator use for >48 hours, acute renal failure, cerebrovascular accident, cardiac arrest requiring cardiopulmonary resuscitation, myocardial infarction, deep vein thrombosis requiring therapy, sepsis, or septic shock, and mortality within 30 days of the procedure [12].

Statistical analysis

Chi-squared tests were used to compare categorical patient characteristics and surgical outcomes across all racial/ethnic groups, and one-way ANOVA tests with post-hoc t-tests were used to compare differences in racial/ethnic groups for continuous variables. All continuous variables met the normality assumption of a one-way ANOVA. Univariate regressions were used to select variables, and any variables with a p<0.25 were included in the multivariable models [13]. Potential confounders were assessed by evaluating changes in the effect estimate upon their inclusion in the multivariable model. Variables that resulted in a >10% change in the odds ratio for the primary exposure were considered confounders and retained in the final model. To assess the potential impact of unmeasured confounders, we performed an E-value analysis, estimating the strength of an unmeasured confounder that would be necessary to fully explain the observed associations. There were no highly collinear baseline factors. Multivariable logistic regression models were used to examine the association between race and 30-day readmission, medical complications, reoperation, and non-home discharge destination while controlling for patient covariates. Multivariable linear regression models were used to examine total hospital length of stay. Adjusted odds ratios (AORs) and 95% confidence intervals (CIs) were calculated. We applied the Bonferroni correction to account for multiple hypothesis testing and reduce the risk of type I error, adjusting the significance threshold accordingly for secondary analyses. Significance was set at p<0.05.

Relative comparisons can be hard to contextualize, especially when the relative rate or a poor outcome is infrequent. Hence, we also considered our results in absolute terms as well. The number needed to expose was calculated for one additional person to be harmed (NNEH) using the AOR and the unexposed event rate. NNEH is analogous to the number needed to harm with adjustment for confounding variables, making it a more appropriate measure in observational studies. Statistical analysis was performed using R software (version 4.1.0, Vienna, Austria), RStudio (version 1.4.1717, Boston, MA), and the tidyverse package [14,15].

## Results

We analyzed patient characteristics of age, sex, BMI, smoking status, ASA physical status classification score, preoperative medical comorbidities, and operative time. Of all 113,340 lumbar spine surgeries meeting inclusion and exclusion criteria, 71.64% (n=81,193) were non-Hispanic White patients, 7.54% (n=8,550) were non-Hispanic Black patients, 2.39% (n=2,708) were non-Hispanic Asian patients, 0.38% (n=429) were non-Hispanic AI/AN patients, 0.20% (n=229) were non-Hispanic NH/PI patients, 6.19% (n=7,015) were Hispanic/Latino patients, and 11.40% (n=12,743) were unknown race and/or ethnicity. The racial/ethnic composition did not differ by year. Non-Hispanic Black and AI/AN patients were more likely to actively smoke, have an ASA ≥3, and have multiple medical comorbidities. In addition, NH/PI patients were more likely to have multiple medical comorbidities (Table 1). Compared to White patients, the unadjusted regression models showed that all minoritized patients were associated with adverse outcomes relative to non-Hispanic White patients (Table 2).

**Table 1:** Patient demographics of each race/ethnicity of lumbar spine procedures from 2017 to 2020 Preliminary ANOVA tests showed significance at p<0.001 for each patient variable. Pair-wise t-tests were done between each minority group and the non-Hispanic White. p-values were calculated for all racial groups. The p-values for the primary groups discussed (AI/AN and NH/PI) are included in this table. BMI: body mass index, ASA: American Society of Anesthesiologists, SD: standard deviation, AI/AN: American Indian/Alaskan Native, NH/PI: Native Hawaiian/Pacific Islander, ANOVA: analysis of variance, ±: plus-or-minus sign

Patient characteristics	Non-Hispanic White % (n) or mean (SD)	Non-Hispanic Black % (n) or mean (SD)	Non-Hispanic Asian % (n) or mean (SD)	Non-Hispanic AI/AN % (n) or mean (SD)	p-value	Non-Hispanic NH/PI % (n) or mean (SD)	p-value	Hispanic/Latino % (n) or mean (SD)
Number	71.64% (81193 total) (43221 male)	7.54% (8550 total) (3959 male)	2.39% (2708 total) (1357 male)	0.38% (429 total) (201 male)	NA	0.20% (229 total) (144 male)	NA	6.19% (7015 total) (3618 male)
Age (y)	60.3 ± 14.6	57.2 ± 12.6	60 ± 16.1	54.6 ± 14.8	<0.001	55.1 ± 16.4	<0.001	54.1 ± 15.1
BMI (kg/m2)	30.8 ± 6.38	32.6 ± 6.81	27.3 ± 5.08	32.4 ± 6.62	<0.001	31.8 ± 7.17	0.01752	31.5 ± 6.21
Operative Time (min)	148 ± 97.8	170 ± 104	150 ± 97.8	134 ± 83.8	0.00637	151 ± 102	0.73081	156 ± 102
Female sex	46.80% (37972)	53.70% (4591)	49.90% (1351)	53.10% (228)	0.01099	37.10% (85)	0.00539	48.40% (3397)
Active smoking status	17.80% (14448)	23.30% (1995)	10.70% (291)	29.40% (126)	<0.001	18.30% (42)	0.83027	16.80% (1180)
ASA class ≥3	48.60% (39499)	54.30% (4644)	34.30% (928)	57.60% (247)	<0.001	46.30% (106)	0.49171	42.00% (2948)
Single comorbidity	38.20% (30991)	39.80% (3404)	34.20% (925)	33.60% (144)	0.09751	35.40% (81)	0.51762	31.00% (2173)
Multiple comorbidities	23.30% (18886)	32.80% (2805)	25.00% (677)	26.10% (112)	0.23034	27.10% (62)	0.23084	24.10% (1694)

**Table 2:** Postoperative outcomes of lumbar spine procedures by race/ethnicity Preliminary ANOVA tests showed significance at p<0.001 for each surgical outcome. Pair-wise t-tests were done between each minority group and the non-Hispanic White. SD: standard deviation, AI/AN: American Indian/Alaskan Native, NH/PI: Native Hawaiian/Pacific Islander, ±: plus-or-minus sign

Outcomes	Non-Hispanic White % (n) or mean (SD)	Non-Hispanic Black % (n) or mean (SD)	Non-Hispanic Asian % (n) or mean (SD)	Non-Hispanic AI/AN % (n) or mean (SD)	p-value	Non-Hispanic NH/PI % (n) or mean (SD)	P-value	Hispanic/Latino % (n) or mean (SD)
Readmission	4.70% (3795)	6.00% (509)	2.90% (79)	7.00% (30)	0.03567	7.90% (18)	0.03567	4.60% (323)
Postoperative complications	8.40% (6789)	11.00% (941)	6.60% (178)	10.30% (44)	0.2745	10.50% (24)	0.35	7.40% (522)
Major complications	2.80% (2274)	4.30% (368)	2.10% (56)	4.00% (17)	0.2216	5.70% (13)	0.0287	2.40% (170)
Minor complications	6.60% (5383)	8.30% (712)	5.40% (146)	7.70% (33)	0.553	7.40% (17)	0.718	5.90% (413)
Reoperation	2.60% (2081)	3.80% (325)	1.80% (50)	3.50% (15)	0.30974	6.10% (14)	0.0034	2.50% (178)
Discharge destination other than home	11.50% (9372)	19.10% (1636)	12.70% (345)	11.20% (48)	0.8209	15.70% (36)	0.1091	12.40% (872)
Mortality	0.20% (168)	0.20% (19)	0.10% (4)	0.00% (0)	0.72	0.90% (2)	0.11	0.20% (14)
Total hospital length of stay	2.41 ± 3.12	3.42 ± 4.11	2.8 ± 3.38	2.41 ± 3.03	0.98397	2.94 ± 3.81	0.02852	2.88 ± 3.84

A multivariable regression was performed to assess the impact of race/ethnicity on patient outcomes while controlling for patient demographic and clinical characteristics. We found that non-Hispanic Black patients had 0.7% higher odds of readmission (AOR: 1.007, 95% CI: 1.002-1.012), 0.9% higher odds of major complication (AOR: 1.009, 95% CI: 1.005-1.013), 0.8% higher odds of reoperation (AOR: 1.008, 95% CI: 1.004-1.012), 6.4% higher odds of non-home discharge (AOR: 1.064, 95% CI: 1.057-1.072), and nearly 1.0 day longer in the hospital (AOR: 1.993, 95% CI: 1.858-2.137) compared to non-Hispanic White patients (Table 3). Hispanic/Latino patients had 2.8% higher odds of non-home discharge (AOR: 1.028, 95% CI: 1.020-1.036) but 1.0% lower odds of any postoperative medical complications (AOR: 0.990, 95% CI: 0.984-0.997) and 0.8% lower odds of minor complications (AOR: 0.992, 95% CI: 0.986-0.997) compared to non-Hispanic White patients (Tables 3-4). In absolute terms, Hispanic patients experience one additional readmission and non-home discharge for every 42 to 1,049 Hispanic patients who undergo a lumbar spine procedure.

**Table 3:** Multivariable regression models of lumbar spine outcomes with race/ethnicity referenced against the non-Hispanic White cohort for reoperation, non-home discharge destination, mortality, and total hospital length of stay Multivariable analysis was used to compare the relationship between race/ethnicity (i.e., non-Hispanic Black, non-Hispanic Asian, non-Hispanic AI/AN, non-Hispanic NH/PI, Hispanic/Latino, and unknown ethnicity) with 30-day postoperative outcomes (i.e., readmission, all postoperative medical complications, major medical complication, minor medical complication, reoperation, and total hospital length of stay). Non-Hispanic White was the reference group. BMI: body mass index, ASA: American Society of Anesthesiologists, AI/AN: American Indian/Alaskan Native, NH/PI: Native Hawaiian/Pacific Islander, CI: confidence interval, AOR: adjusted odds ratio

Patient characteristics and comorbidities	Reoperation (AOR (95% CI))	p-value	Non-home discharge destination (AOR (95% CI))	p-value	Mortality (AOR (95% CI))	p-value	Total hospital length of stay (AOR (95% CI))	p-value
Age (y)	1.000 (1.000-1.000)	0.022	1.004 (1.003-1.004)	<0.001	1.000 (1.000-1.000)	<0.001	1.014 (1.013-1.016)	<0.001
Female sex	1.004 (1.002-1.006)	<0.001	1.039 (1.035-1.043)	<0.001	0.999 (0.999-1.000)	0.123	1.418 (1.368-1.471)	<0.001
Non-Hispanic Black	1.008 (1.004-1.012)	<0.001	1.064 (1.057-1.072)	<0.001	1.000 (0.999-1.001)	0.860	1.993 (1.858-2.137)	<0.001
Non-Hispanic Asian	0.994 (0.988-1.001)	0.077	1.018 (1.006-1.030)	0.003	0.999 (0.998-1.001)	0.271	1.510 (1.340-1.702)	<0.001
Non-Hispanic AI/AN	1.008 (0.993-1.024)	0.290	1.017 (0.988-1.047)	0.252	0.998 (0.994-1.002)	0.009	1.186 (0.883-1.593)	0.257
Non-Hispanic NH/PI	1.035 (1.014-1.057)	0.001	1.063 (1.022-1.106)	0.002	1.007 (1.001-1.013)	0.328	1.846 (1.234-2.763)	0.003
Hispanic/Latino	0.999 (0.995-1.003)	0.557	1.028 (1.020-1.036)	<0.001	1.000 (0.999-1.001)	0.061	1.666 (1.544-1.798)	<0.001
Unknown ethnicity	1.004 (1.001-1.008)	0.008	0.986 (0.980-0.992)	<0.001	1.000 (0.999-1.001)	0.272	2.245 (2.108-2.390)	<0.001
Unknown race	1.000 (0.994-1.007)	0.882	1.018 (1.006-1.031)	0.003	0.999 (0.998-1.001)	0.055	1.164 (1.029-1.316)	0.015
BMI (kg/m2)	1.000 (1.000-1.000)	0.003	1.000 (1.000-1.001)	0.085	1.000 (1.000-1.000)	<0.001	0.989 (0.986-0.992)	<0.001
Operative time (min)	1.001 (1.001-1.001)	<0.001	1.010 (1.010-1.010)	<0.001	1.000 (1.000-1.000)	<0.001	1.199 (1.195-1.202)	<0.001
ASA class ≥3	1.006 (1.004-1.008)	<0.001	1.056 (1.052-1.060)	<0.001	1.002 (1.001-1.003)	<0.001	1.819 (1.744-1.897)	<0.001
Smoking status	1.004 (1.002-1.007)	0.00117	0.994 (0.989-0.998)	0.009	1.000 (1.000-1.001)	0.838	1.067 (1.016-1.120)	0.009
One comorbidity	1.003 (1.000-1.005)	0.033	0.990 (0.986-0.995)	<0.001	1.000 (0.999-1.001)	0.922	1.073 (1.025-1.124)	0.003
Multiple comorbidities	1.009 (1.006-1.012)	<0.001	1.053 (1.048-1.059)	<0.001	1.003 (1.002-1.004)	<0.001	1.876 (1.775-1.984)	<0.001

**Table 4:** Multivariable regression models of lumbar spine outcomes with race/ethnicity referenced against the non-Hispanic White cohort for readmission and postoperative (major and minor) complications Multivariable analysis was used to compare the relationship between race/ethnicity (i.e., non-Hispanic Black, non-Hispanic Asian, non-Hispanic AI/AN, non-Hispanic NH/PI, Hispanic/Latino, and unknown ethnicity) with 30-day postoperative outcomes (i.e., readmission, all postoperative medical complications, major medical complication, minor medical complication, reoperation, and total hospital length of stay). Non-Hispanic White was the reference group. BMI: body mass index, ASA: American Society of Anesthesiologists, AI/AN: American Indian/Alaskan Native, NH/PI: Native Hawaiian/Pacific Islander, CI: confidence interval, AOR: adjusted odds ratio

Patient characteristics and comorbidities	Readmission (AOR (95% CI))	p-value	Postoperative complications (AOR (95% CI))	p-value	Postoperative major complications (AOR (95% CI))	p-value	Postoperative minor complications (AOR (95% CI))	p-value
Age (y)	1.000 (1.000-1.000)	<0.001	1.001 (1.000-1.001)	<0.001	1.000 (1.000-1.000)	<0.001	1.001 (1.000-1.001)	<0.001
Female sex	1.005 (1.003-1.008)	<0.001	1.023 (1.020-1.027)	<0.001	1.000 (0.998-1.002)	0.951	1.024 (1.022-1.027)	0.005
Non-Hispanic Black	1.007 (1.002-1.012)	0.003	1.005 (1.000-1.012)	0.072	1.009 (1.005-1.013)	<0.001	0.999 (0.993-1.004)	0.003
Non-Hispanic Asian	0.986 (0.978-0.994)	<0.001	0.982 (0.972-0.992)	<0.001	0.995 (0.989-1.001)	0.119	0.986 (0.977-0.995)	<0.001
Non-Hispanic AI/AN	1.023 (1.003-1.043)	0.026	1.030 (1.004-1.056)	0.023	1.012 (0.997-1.028)	0.123	1.021 (0.999-1.045)	0.192
Non-Hispanic NH/PI	1.033 (1.006-1.062)	0.018	1.025 (0.990-1.060)	0.167	1.029 (1.007-1.051)	0.009	1.011 (0.981-1.043)	0.464
Hispanic/Latino	1.001 (0.996-1.007)	0.606	0.990 (0.984-0.997)	0.003	0.997 (0.993-1.001)	0.157	0.992 (0.986-0.997)	0.006
Unknown ethnicity	0.994 (0.990-0.999)	0.009	1.010 (1.004-1.015)	<0.001	1.001 (0.998-1.005)	0.472	1.008 (1.003-1.012)	<0.001
Unknown race	1.004 (0.996-1.013)	0.317	0.998 (0.987-1.008)	0.668	1.004 (0.997-1.010)	0.271	0.994 (0.985-1.003)	0.097
BMI (kg/m2)	1.000 (1.000-1.000)	0.087	1.000 (0.999-1.000)	0.008	1.000 (1.000-1.000)	0.029	0.999 (0.999-1.000)	<0.001
Operative time (min)	1.001 (1.001-1.002)	<0.001	1.012 (1.012-1.012)	<0.001	1.002 (1.002-1.002)	<0.001	1.011 (1.011-1.011)	<0.001
ASA class ≥3	1.018 (1.015-1.021)	<0.001	1.026 (1.022-1.029)	<0.001	1.013 (1.011-1.015)	<0.001	1.019 (1.016-1.022)	<0.001
Smoking status	1.008 (1.004-1.011)	<0.001	1.003 (0.998-1.007)	0.229	1.003 (1.001-1.006)	0.016	0.999 (0.996-1.003)	<0.001
One comorbidity	1.004 (1.001-1.007)	0.019	1.006 (1.002-1.010)	0.003921	1.004 (1.002-1.007)	<0.001	1.004 (1.000-1.007)	<0.001
Multiple comorbidities	1.024 (1.020-1.028)	<0.001	1.037 (1.032-1.042)	<0.001	1.022 (1.019-1.025)	<0.001	1.027 (1.023-1.031)	<0.001

Non-Hispanic Asian patients had 1.8% higher odds of non-home discharge destination (AOR: 1.018, 95% CI: 1.006-1.030) and over 0.5 days longer in the hospital (AOR: 1.510, 95% CI: 1.340-1.702), but 1.4% lower odds of readmission (AOR: 0.986, 95% CI: 0.978-0.994), 1.8% lower odds of any postoperative medical complications (AOR: 0.982, 95% CI: 0.972-0.992), 1.4% lower odds of minor complications (AOR: 0.986, 95% CI: 0.977-0.995), and 0.6% lower odds of reoperation (AOR: 0.994, 95% CI: 0.988-1.001), compared to non-Hispanic White patients (Tables 3-4). In absolute terms, Asian patients experience one non-home discharge for every 65 Asian patients who undergo a lumbar spine procedure.

Non-Hispanic AI/AN patients had 2.3% higher odds of readmission (AOR: 1.023, 95% CI: 1.003-1.043) and 3.0% higher odds of any postoperative medical complications (AOR: 1.030, 95% CI: 1.004-1.056) compared to non-Hispanic White patients (Tables 3-4). In absolute terms, AI/AN patients experience one readmission, any one medical complication, reoperation, and non-home discharge for every 38 to 131 AI/AN patients that undergo a lumbar spine procedure.

Non-Hispanic NH/PI patients had 3.3% higher odds of readmission (AOR: 1.033, 95% CI: 1.006-1.062), 2.9% higher odds of major complication (AOR: 1.029, 95% CI: 1.007-1.051), 3.5% higher odds of reoperation (AOR: 1.035, 95% CI: 1.014-1.057), 6.3% higher odds of non-home discharge destination (AOR: 1.063, 95% CI: 1.022-1.106), and 0.85 days longer in the hospital (AOR: 1.846, 95% CI: 1.234-2.763) compared to non-Hispanic White patients (Tables 3-4). In absolute terms, NH/PI patients experience one readmission, major complication, reoperation, and non-home discharge for every 31 to 38 NH/PI patients that undergo a lumbar spine procedure.

## Discussion

Our study builds upon prior research by highlighting outcomes of other minority groups, including NH/PI and AI/AN populations. Importantly, we found that non-Hispanic AI/AN and NH/PI patients had higher complication rates and reoperation when compared to non-Hispanic White patients. Hispanic/Latino and Asian patients were more likely to have increased LOS and non-home discharge but had lower rates of any complication when compared to White patients. This study is an important step in recognizing other racial minority groups and how they may differ from the majority and from each other.

Our analysis focused on AI/AN and NH/PI populations. Multivariable regression models showed that non-Hispanic AI/AN and NH/PI patients faced higher odds of readmission and complications compared to White patients in lumbar spine procedures. Notably, NH/PI patients had the highest odds of readmission, postoperative major complications, and reoperation compared to all minority groups, with one adverse outcome for every 31-38 patients. These higher odds of readmission and complications are similar to those found in more heavily researched Black populations [16,17]. In both AI/AN and NH/PI populations, we observed increased odds of readmission and any complication. This may be linked to the increased risk of superficial surgical site infections and inpatient hospitalization in AI/AN patients previously reported [10]. Furthermore, both AI/AN and NH/PI patients were more likely to have a higher BMI and a greater number of comorbidities, which have been associated with adverse outcomes after lumbar surgery [18-20]. Compared to White patients, AI/AN patients had a higher rate of smoking, which is linked to poorer outcomes [21,22]. While prior studies have shown similar outcomes between Asian and White patients following spinal surgery, few have investigated specific groups such as AI/AN and NH/PI populations [23]. One study, in a cohort of patients within the NSQIP who underwent surgery, found that both AI/AN and NH/PI patients had poorer outcomes when compared to other racial groups, highlighting the existing, and understudied, health disparities within this patient population.

While the primary focus of the current study is to analyze racial disparities between AI/AN and NH/PI patients compared to other groups, we found disparities within other racial groups as well. Consistent with previous literature, we found that Black patients have higher odds of readmission, postoperative major complications, and reoperation compared to White patients [16,24,25]. Contrary to some previous literature, Hispanic and Latino patients in our study had lower rates of postoperative complications [24]. However, these findings support other studies that found Hispanic patients to have similar outcomes when compared to White patients. A 2022 study by Volaski et al. investigating disparities following spinal surgery found Hispanic patients to have no difference in discharge disposition or 30-day readmission rates [26]. Our understanding of the healthcare outcomes in Hispanic and Latino populations would benefit from an increased focus on the factors contributing to improved complication rates. Additionally, we found non-NH/PI and AI/AN Asian patients have longer lengths of stay than White patients, with lower odds of readmission, complications, and reoperation. Improved outcomes in this group have been poorly explored, with some studies finding minimal or no difference between non-NH/PI and AI/AN Asian patients and White patients [23]. Additionally, while we found an increased prevalence of ASA class >3 in Black and AI/AN populations, it was not statistically significant in multivariable analysis. This may suggest that non-clinical factors like socioeconomic status (SES) and cultural beliefs may have a more profound impact than initially thought.

Racial disparities in healthcare exist for various reasons, including patient, provider, and systemic factors [27-29]. Racial and ethnic minoritized populations have worse complication rates in the management of degenerative lumbar spinal changes when compared to their White counterparts [30,31]. Racial minority patients have a greater risk of developing postoperative complications, readmission, and mortality following spine surgery [10,16,17,32]. High rates of complications may stem from a higher number of comorbidities in Black patients resulting from a myriad of predisposing factors [33,34]. However, when we controlled for patient factors commonly associated with postoperative complications and poor outcomes, Black, AI/AN, and NH/PI populations remained independently associated with increased complication rates and poorer outcomes overall. Notably, Asian and Hispanic populations were found to have lower rates of complications when compared to a non-Hispanic White population. There may be other non-clinical factors that are not captured by our variables that influence these associations. The reasoning for these disparities is not fully understood. However, recent literature suggests that physician recommendations, socioeconomic factors, cultural beliefs, mistrust of the healthcare system, risk aversion to newer procedures, and physicians’ implicit biases are the root causes of racial disparities across multiple surgical disciplines [35-37].

There may also be differences in the way racial and ethnic minoritized patients interact with the healthcare system that may explain our study results. In prior literature, compared with other racial groups, Black patients were less likely to choose operative management and more likely to show lower satisfaction scores [38-40]. This may be partly due to mistrust between patients and healthcare professionals. While this was not specifically assessed in the AI/AN and NH/PI populations, the history of historical trauma in both populations could contribute to mistrust [41,42]. Mistrust of Western medicine and utilization of traditional native healers, a well-described phenomenon, may also play a role in perpetuating disparities by decreasing surgery utilization [43-45]. Health literacy among NH/PI and AI/AN populations remains low [46]. The large discrepancy between racial and ethnic minority numbers validated in census figures and the relatively low number in spinal procedures suggests that many patients do not have institutional support or resources. There is a great need for improved outreach to ensure that patients who need to be treated can get the operations they need. Strategies to optimize care and improve outreach for minorities include educating physicians on implicit bias and the development of modified decision aids [47,48].

Limitations

This study has several limitations. First, the ACS-NSQIP database provides a large but surface-level overview of patient outcomes. This study should be replicated with more focused neurosurgical datasets to increase the applicability of these results. Additionally, ACS-NSQIP does not include patient SES, a well-known factor associated with differences in health and spinal surgical outcomes [25,49]. Past research has suggested non-clinical factors that may affect outcomes. Additional study of socioeconomic factors and cultural beliefs in a smaller, highly focused study may provide valuable insights. Finally, the geographic distribution of different races and ethnicities is not homogenous across the entirety of the United States. This is particularly true for NH/PI and AI/AN populations. Differences in location, socio-political environment, and other factors may contribute to spurious correlations about the apparent association of race and ethnicity on spinal surgical outcomes.

## Conclusions

Racial disparity research has grossly overlooked Hispanic, Asian, AI/AN, and NH/PI groups. Race and ethnicity are associated with adverse outcomes, specifically higher risks for readmission, reoperation, and complications in AI/AN and NH/PI groups. Thus, greater attention should be given to these populations, which have traditionally not been included in studies or inappropriately grouped into a single “other” racial group. Future studies should consider disaggregating broad racial classifications to develop a more tailored approach to reduce adverse outcomes.
